# RETIREMENT TRANSITIONS AND COMPLETED SUICIDE DURING RECOVERY FROM THE GREAT RECESSION: EVIDENCE FROM THE NVDRS

**DOI:** 10.1093/geroni/igac059.1472

**Published:** 2022-12-20

**Authors:** Briana Mezuk, Aparna Ananthasubramaniam, Linh Dang, David Jurgens

**Affiliations:** University of Michigan, School of Public Health, Ann Arbor, Michigan, United States; University of Michigan, Ann Arbor, Michigan, United States; University of Michigan, Ann Arbor, Michigan, United States; University of Michigan, Ann Arbor, Michigan, United States

## Abstract

One million older Americans retire annually. While these transitions are not generally associated with poor mental health, the broader macro-economic context in which retirement transitions take place may shape how they relate to mental health. The objective of this study was to use state-of-the-art natural language processing (NLP) to develop a model to identify retirement transitions from textual data in the National Violent Death Reporting System (NVDRS), and to use that model to examine how the number of suicides related to retirement transitions changed during the recovery from the Great Recession. Data come from the NVDRS (2003 - 2018, n=62,165), a state-based registry of suicide deaths. We used RoBERTa to train a NLP model to identify retirement transitions (e.g., recent retirement, anticipated retirement, unable to retire despite wanting to) from 1,291 annotated sentences from NVDRS text narratives of suicide decedents aged ≥55 (model performance: F1=0.92). Applying this model, 19.35 of every 1,000 suicides among decedents aged ≥55 years mentioned a retirement transition. Decedent characteristics associated with retirement transitions were younger age (< 75 years), having a college education and experiencing financial problems. The probability that a narrative referenced a retirement transition increased 1.495-fold during the Great Recession (2007 - 2009) and declined during recovery (2009-2016) before beginning to increase again. Findings illustrate the utility of NLP methods to identify workforce transitions from NVDRS narratives, and further understanding the impact of macro contextual events like the Great Recession on population mental health.

